# Ocular instillation of conditioned medium from mesenchymal stem cells is effective for dry eye syndrome by improving corneal barrier function

**DOI:** 10.1038/s41598-023-40136-2

**Published:** 2023-08-11

**Authors:** Tsutomu Imaizumi, Ryuhei Hayashi, Yuji Kudo, Xiaoqin Li, Kaito Yamaguchi, Shun Shibata, Toru Okubo, Tsuyoshi Ishii, Yoichi Honma, Kohji Nishida

**Affiliations:** 1 Department of Stem Cells and Applied Medicine, Osaka University Graduate School of Medicine, Suita, Osaka 565-0871 Japan; 2 Basic Research Development Division, ROHTO Pharmaceutical, Ikuno-ku, Osaka 544-8666 Japan; 3 Department of Ophthalmology, Osaka University Graduate School of Medicine, Suita, Osaka 565-0871 Japan; 4 Institute for Open and Transdisciplinary Research Initiatives, Osaka University, Osaka, Osaka 565-0871 Japan; 5 Department of Informative Genetics, Tohoku University Graduate School of Medicine, Sendai, Miyagi 980-8575 Japan

**Keywords:** Mesenchymal stem cells, Corneal diseases

## Abstract

Dry eye syndrome (DES) is a chronic ocular disease that induces epithelial damage to the cornea by decreasing tear production and quality. Adequate treatment options have not been established for severe DES such as Sjogren’s syndrome due to complicated pathological conditions. To solve this problem, we focused on the conditioned medium of human adipose-derived mesenchymal stem cells (hAdMSC-CM), which have multiple therapeutic properties. Here, we showed that hAdMSC-CM suppressed Benzalkonium Chloride (BAC)-induced cytotoxicity and inflammation in human corneal epithelial cells (hCECs). In addition, hAdMSC-CM increased the expression level and regulated the localisation of barrier function-related components, and improved the BAC-induced barrier dysfunction in hCECs. RNA-seq analysis and pharmacological inhibition experiments revealed that the effects of hAdMSC-CM were associated with the TGFβ and JAK-STAT signalling pathways. Moreover, in DES model rats with exorbital and intraorbital lacrimal gland excision, ocular instillation of hAdMSC-CM suppressed corneal epithelial damage by improving barrier dysfunction of the cornea. Thus, we demonstrated that hAdMSC-CM has multiple therapeutic properties associated with TGFβ and JAK-STAT signalling pathways, and ocular instillation of hAdMSC-CM may serve as an innovative therapeutic agent for DES by improving corneal barrier function.

## Introduction

Dry eye syndrome (DES) is a chronic ocular disease that is recognised worldwide. The number of patients with DES is increasing rapidly due to various factors, such as extensive time spent on screens in this era of information technology and aging^1,2^. DES is characterised by decreased tear production or tear quality, which leads to corneal damage^1,2^. Several treatments, such as artificial tear solutions, lacrimal secretion stimulants, and anti-inflammatory agents are currently being used for DES, but adequate treatment options have not been established for severe DES, such as Sjogren’s syndrome, which is an autoimmune disease. This is due to complicated pathological conditions that include epithelial damage, inflammation, and barrier dysfunction of the cornea^3–5^. Therefore, to address this issue, the development of new therapeutic agents with multiple properties is required.

Human mesenchymal stem cells (hMSCs) are multipotent stem cells with the ability to self-replicate and differentiate^6^. hMSCs also possess multiple therapeutic properties, such as anti-inflammatory effects, anti-apoptotic activity, and wound healing in various tissues, by secreting multiple factors^7,8^. Therefore, hMSCs are believed to function as a new therapeutic agent for various diseases, including inflammatory diseases, neurological disorders, and fibrosis^7,8^. In addition, hMSCs are isolated from various tissues, including the bone marrow, umbilical cord blood, placenta, and adipose tissue^9,10^. Furthermore, hMSCs have their own characteristics depending on the isolated tissues, and adipose-derived MSCs (hAdMSCs) are relatively easy to isolate; therefore, hAdMSCs are considered useful for practical applications of cell therapy^9,10^.

The hAdMSCs are believed to have potential applications in corneal diseases, retinal diseases, and optic neuropathy^11^. The conditioned medium of hAdMSCs (hAdMSC-CM) contains secreted factors from hAdMSCs, has several therapeutic properties, and is expected to function as a new therapeutic agent^12,13^. Additionally, hAdMSC-CM is cheaper to obtain and is safer to use as compared to the hAdMSC therapy^12,13^. In a previous study, we revealed that hAdMSC-CM attenuated epithelial-mesenchymal transition in human corneal epithelial cells (hCECs) and may be a useful treatment for corneal diseases^14^. It has been reported that secreted factors from hAdMSCs suppress cytotoxicity and inflammation in hCECs, and ocular instillation of these secreted factors suppresses epithelial damage and inflammation of the cornea in an in vivo DES model^15–17^. However, the effects of hAdMSC-CM on DES have not been sufficiently investigated; in particular, the barrier function has not been revealed.

Ocular instillation of hAdMSC-CM has not been evaluated in an in vivo DES model with corneal epithelial damage caused by decreased tear production, which is characteristic of DES. To study DES, there are several established animal models that can be used, such as scopolamine-treated, high air flow environment, and lacrimal gland excision models^18–20^. Notably, animal models with exorbital and intraorbital lacrimal gland excision (LG-Ex) are clinically similar to DES, which shows corneal epithelial damage due to decreased tear production^20^.

Therefore, in this study, we evaluated the effects of hAdMSC-CM on complex pathologies of DES, such as cytotoxicity, inflammation, and barrier dysfunction of hCECs, as well as the efficacy of ocular instillation of hAdMSC-CM in a rat LG-Ex DES model.

## Results

### Effects of hAdMSC-CM on benzalkonium chloride (BAC)-induced cytotoxicity and inflammation in hCECs

**Figure 1:**
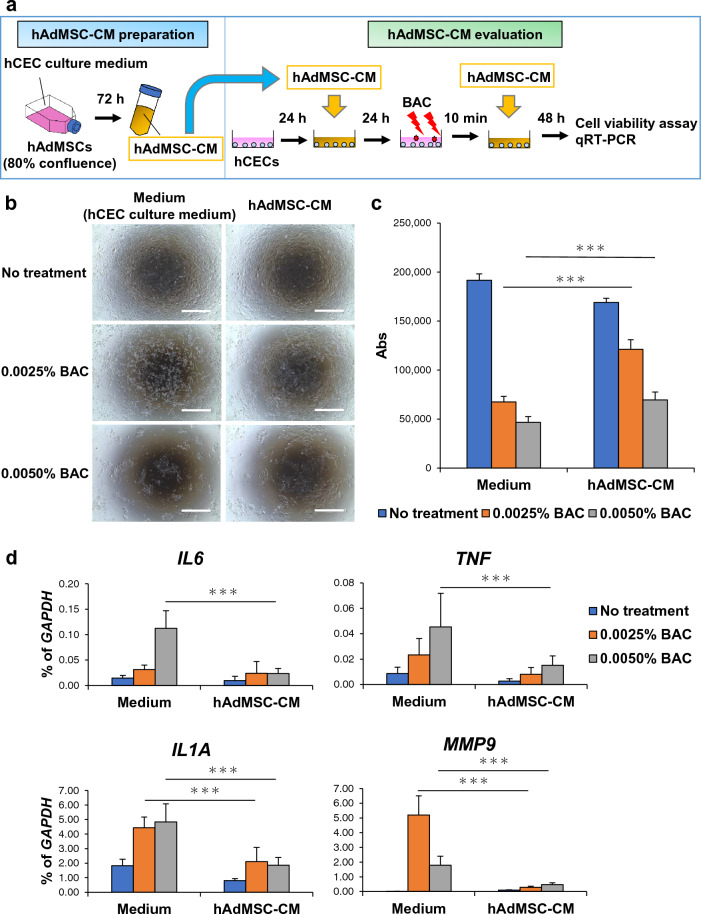
hAdMSC-CM suppresses BAC-induced cytotoxicity and inflammation in hCECs. (**a**) Schematic representation of hAdMSC-CM preparation using hCEC culture medium and the evaluation of hAdMSC-CM on BAC-induced cell cytotoxicity and inflammation in hCECs. (**b**) Phase images of hCECs 2 days after treatment with or without BAC. Scale bar, 1000 µm. *n* = 9 biological replicates. (**c**) Cell viability assay of hCECs 2 days after treatment with or without BAC. The results are presented as the mean ± SD; *n* = 9 biological replicates. ***p < 0.001. (**d**) Expression levels of inflammation-related genes in hCECs 2 days after treatment with or without BAC. The results are presented as the mean ± SD; *n* = 9 biological replicates. ***p < 0.001. hAdMSC-CM; conditioned medium of human adipose-derived mesenchymal stem cells, BAC; benzalkonium chloride, hCECs; human corneal epithelial cells.

DES is associated with cytotoxicity, inflammation, and barrier dysfunction in the cornea^4,5^. In addition, BAC, a widely used preservative in eye drops, induces cytotoxicity, inflammation, and barrier dysfunction of hCECs. Thus, BAC-treated hCECs are used to study DES^21–24^. We first evaluated the effects of hAdMSC-CM on BAC-induced cytotoxicity and inflammation in hCECs (Fig. 1a). Before preparing hAdMSC-CM, we confirmed that hAdMSCs showed typical fibroblast-like morphology and expressed CD29, CD73, CD90, and CD105 as positive markers of hMSCs and lacked expression of CD34 and CD45 as negative markers of hMSCs (Supplementary Fig. S1a, S1b).

Subsequently, two types of hAdMSC-CM were collected from hMSC and hCEC culture medium. hCECs were cultured in both types of hAdMSC-CM 1 day before and immediately after BAC stimulation. 2 days after cultivation of hCECs, their morphology could not be maintained with hAdMSC-CM derived from hMSC culture medium (hAdMSC-CM (m)) (Supplementary Fig. S2a, S2b). However, hAdMSC-CM derived from hCEC culture medium adequately maintained hCEC morphology, suggesting that hAdMSC-CM based on hCEC culture medium perform better for hCEC treatments; based on this finding, we focused on the effects of using hCEC culture medium-derived hAdMSC-CM (Fig. 1a,b). 2 days after stimulating hCECs with BAC, a cell viability assay and qRT-PCR were performed. These experiments showed that BAC-mediated cytotoxicity against hCECs increased in a concentration-dependent manner, which was attenuated by hAdMSC-CM (Fig. 1b,c). Furthermore, the expression levels of inflammation-related genes, such as *IL6*, *IL1A*, *TNF*, and *MMP9*, in hCECs, increased with BAC, and this effect was suppressed by hAdMSC-CM (Fig. 1d). These results indicate that hAdMSC-CM could suppress BAC-induced cytotoxicity and inflammation in hCECs.

### Effects of hAdMSC-CM on BAC-induced barrier dysfunction in hCECs

**Figure 2:**
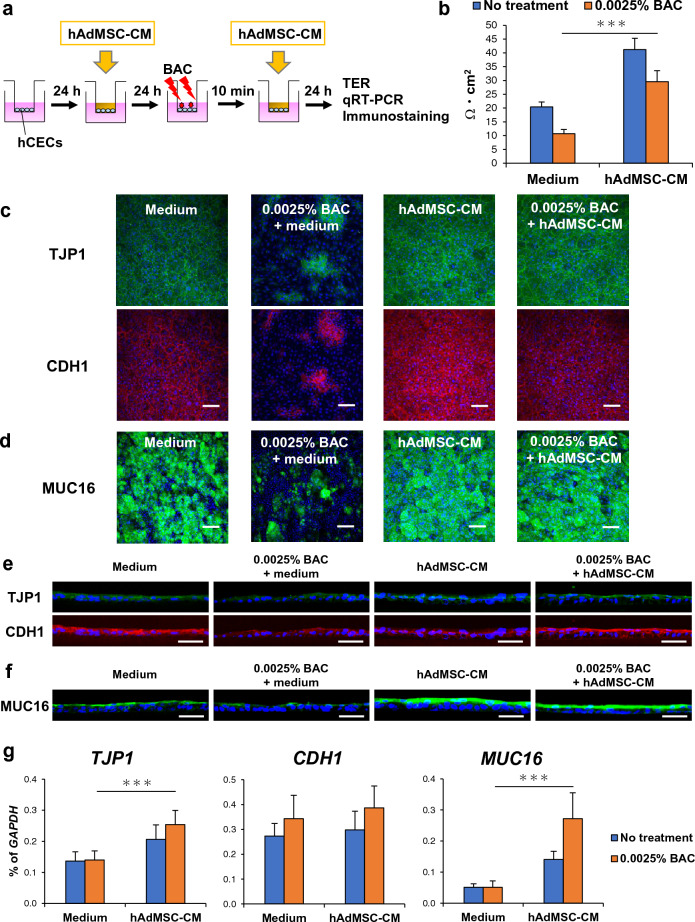
hAdMSC-CM improves BAC-induced barrier dysfunction of hCECs. (**a**) Schematic representation of evaluation of hAdMSC-CM on BAC-induced barrier dysfunction of hCECs. (**b**) Quantification of TER in hCECs 1 day after treatment with or without BAC. The results are presented as the mean ± SD; *n* = 12 biological replicates. ***p < 0.001. (**c**) Immunostaining images of TJP1 and CDH1 in hCECs 1 day after treatment with or without BAC. Scale bar, 100 µm. *n* = 6 biological replicates. (**d**) Immunostaining images of MUC16 in hCECs 1 day after treatment with or without BAC. Scale bar, 100 µm. *n* = 4 biological replicates. (**e**) Immunostaining images of TJP1 and CDH1 in a thin section of hCECs 1 day after treatment with or without BAC. Scale bar, 50 µm. *n* = 4 biological replicates. (**f**) Immunostaining images of MUC16 in a thin section of hCECs 1 day after treatment with or without BAC. Scale bar, 50 µm. *n* = 4 biological replicates. (**g**) Expression levels of barrier function-related genes in hCECs 1 day after treatment with or without BAC. The results are presented as the mean ± SD; *n* = 12 biological replicates. ***p < 0.001.TER; transepithelial electrical resistance.

Next, we evaluated the effects of hAdMSC-CM on BAC-induced barrier dysfunction in hCECs (Fig. 2a). To examine the barrier function, hCECs were cultivated on the cell culture insert in almost confluent cells, which formed sufficient cell junctions. hCECs were cultured in hAdMSC-CM, which was provided only from the apical side of hCECs. The cultures were performed 1 day before and immediately after stimulating hCECs with BAC. 1 day after cultivation of hCECs, the transepithelial electrical resistance (TER) of hCECs, which is an important indicator of cell junctions, was measured. The TER appeared to decrease with BAC (Fig. 2b). However, hAdMSC-CM increased the TER of hCECs, indicating that hAdMSC-CM enhanced the cell junctions and barrier function of hCECs (Fig. 2b). Moreover, we examined barrier function-related components using immunostaining and qRT-PCR. Immunostaining of hCECs showed that the expression levels of barrier function-related proteins, such as TJP1, CDH1, and MUC16, appeared to decrease with BAC, and BAC disrupted the localisation of TJP1 at cell–cell contacts (Fig. 2c–f). In contrast, hAdMSC-CM increased the expression levels of TJP1, CDH1, and MUC16 and promoted the localisation of TJP1 to cell–cell contacts (Fig. 2c–f). Similarly, the expression levels of barrier function-related genes, such as *TJP1* and *MUC16*, in hCECs were increased by hAdMSC-CM (Fig. 2g). These results show that hAdMSC-CM could improve BAC-induced barrier dysfunction in hCECs.

### Investigating the mechanism underlying effects of hAdMSC-CM on BAC-induced cytotoxicity, inflammation, and barrier dysfunction in hCECs

**Figure 3:**
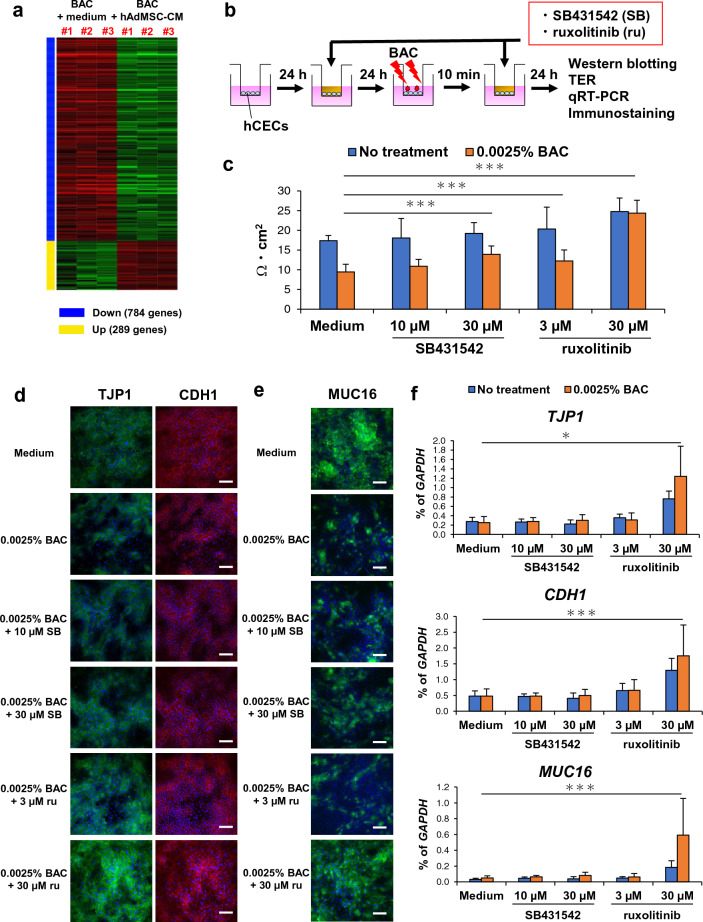
BAC-induced barrier dysfunction of hCECs is improved by inhibition of TGFβ and JAK-STAT signalling pathways. (**a**) Heatmap of differentially expressed genes in hCECs with BAC + medium or BAC + hAdMSC-CM. *n* = 3 biological replicates. (**b**) Schematic representation of evaluation of SB431542 and Ruxolitinib on BAC-induced barrier dysfunction of hCECs. (**c**) Quantification of TER in hCECs 1 day after treatment with or without BAC. The results are presented as the mean ± SD; *n* = 14 biological replicates. ***p < 0.001. (**d**) Immunostaining images of TJP1 and CDH1 in hCECs 1 day after treatment with or without BAC. Scale bar, 100 µm. *n* = 4 biological replicates. (**e**) Immunostaining images of MUC16 in hCECs 1 day after treatment with or without BAC. Scale bar, 100 µm. *n* = 4 biological replicates. (**f**) Expression levels of barrier function-related genes in hCECs 1 day after treatment with or without BAC. The results are presented as the mean ± SD; *n* = 8 biological replicates. *p < 0.05 and ***p < 0.001.

We investigated how hAdMSC-CM improved BAC-induced cytotoxicity, inflammation, and barrier dysfunction in hCECs using RNA-seq analysis. We analysed a heatmap plot of differentially expressed genes in hCECs treated with BAC + medium or BAC + hAdMSC-CM, and we selected the top 30 genes with significant fold reductions in expression levels due to hAdMSC-CM. These included a few genes related to TGFβ and JAK-STAT signalling pathways (Fig. 3a, Supplementary Fig. S3 and Table 1).

**Table 1:** The top 30 genes with significant fold reductions in expression levels due to hAdMSC-CM.

	Symbol	log2 Fold change	Adj. *P* val	JAK-STAT	TGFβ
1	*KRT27*	− 7.18360	2.54E−24		
2	*RPTN*	− 6.15397	1.63E−62		
3	*CYP1A1*	− 5.75414	9.67E−155	**〇**	**〇**
4	*PCDH18*	− 5.29645	1.44E−04		**〇**
5	*BST2*	− 5.28682	5.69E−16	**〇**	**〇**
6	*CD36*	− 5.18155	1.92E−12	**〇**	**〇**
7	*POSTN*	− 5.17511	9.01E−77	**〇**	**〇**
8	*ASPRV1*	− 4.91622	1.54E−14		
9	*CXCL10*	− 4.90288	7.75E−34	**〇**	**〇**
10	*FABP4*	− 4.61846	1.44E−45	**〇**	**〇**
11	*CXCL11*	− 4.54216	3.56E−21	**〇**	**〇**
12	*NUF2*	− 4.47057	2.47E−14	**〇**	
13	*MSMB*	− 4.37828	3.57E−15		**〇**
14	*CPXM2*	− 4.32554	3.01E−67		**〇**
15	*SSC5D*	− 4.27465	1.36E−09		
16	*NXF3*	− 4.25167	3.03E−12		**〇**
17	*DSC1*	− 4.24198	1.36E−34	**〇**	**〇**
18	*KRT3*	− 4.22884	2.07E−73		**〇**
19	*FCRLA*	− 4.21421	3.88E−15		**〇**
20	*CNFN*	− 4.09824	8.91E−44		
21	*AC067930.6*	− 4.08640	3.99E−03		
22	*CAPN8*	− 4.07461	1.80E−38		
23	*IFI44L*	− 4.06611	7.77E−16	**〇**	**〇**
24	*CLEC7A*	− 4.01951	6.11E−81	**〇**	**〇**
25	*LRRC17*	− 4.01841	4.37E−46		
26	*THBS2*	− 3.88031	2.38E−68	**〇**	**〇**
27	*H2BC9*	− 3.87884	2.45E−24		
28	*SPRR1A*	− 3.82991	1.46E−212	**〇**	**〇**
29	*KIAA0319*	− 3.81703	1.03E−12	**〇**	**〇**
30	*ANO3*	− 3.76785	1.08E−11	**〇**	**〇**

Subsequently, we evaluated BAC-induced cytotoxicity and inflammation in hCECs using inhibitors of TGFβ (SB431542) and JAK-STAT signalling pathways (ruxolitinib) (Supplementary Fig. S4a). Cell viability assays and qRT-PCR were performed, and we observed that BAC-induced cytotoxicity of hCECs was attenuated by SB431542 and ruxolitinib (Supplementary Fig. S4b). Moreover, the expression levels of inflammation-related genes, such as *IL6*, *IL1A*, *TNF*, and *MMP9*, in hCECs were suppressed by SB431542 and ruxolitinib; in particular, SB431542 was more effective (Supplementary Fig. S4c). Next, we evaluated BAC-induced barrier dysfunction of hCECs using SB431542 and ruxolitinib, and the experiments showed that TER of hCECs increased due to SB431542 and ruxolitinib; in particular, ruxolitinib was more effective (Fig. 3b,c). In addition, immunostaining of hCECs showed that the expression levels of barrier function-related proteins, such as TJP1 and MUC16, were increased by ruxolitinib, and localisation of TJP1 at cell–cell contacts was promoted by ruxolitinib (Fig. 3d,e). Similarly, the expression levels of barrier function-related genes, such as *TJP1*, *CDH1*, and *MUC16*, in hCECs were increased by ruxolitinib (Fig. 3f). In these experiments, we confirmed that SB431542 and ruxolitinib suppressed pSMAD2 and pSTAT1 in hCECs by western blotting, and cell death in hCECs was largely not induced at the concentrations used in this experiment by TUNEL assay (Supplementary Fig. S5, S6). Our data suggest that BAC-induced cytotoxicity, inflammation, and barrier dysfunction of hCECs were improved by inhibition of TGFβ and JAK-STAT signalling pathways, and the effects of hAdMSC-CM were associated with TGFβ and JAK-STAT signalling pathways.

### Evaluation of ocular instillation of hAdMSC-CM on the cornea in a DES rat model with exorbital and intraorbital lacrimal gland excision

**Figure 4:**
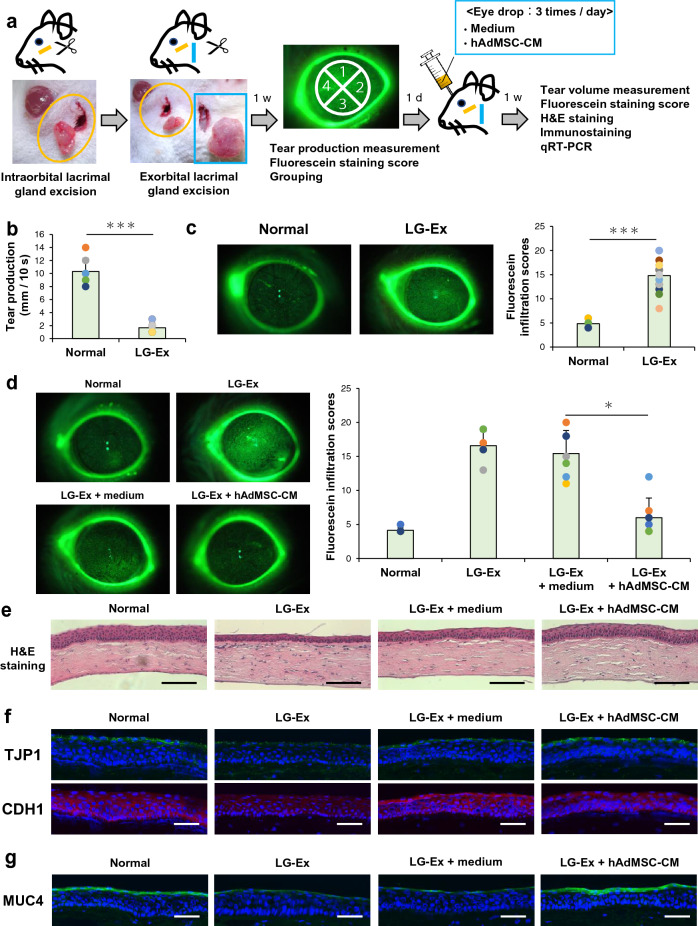
Ocular instillation of hAdMSC-CM suppresses corneal epithelial damage in lacrimal gland excised (LG-Ex) rats by improving barrier dysfunction of the cornea. (**a**) Schematic representation of exorbital and intraorbital LG-Ex rats, ocular instillation of hAdMSC-CM, and evaluation of cornea. (**b**) Measurement of tear production in SD (Normal) and LG-Ex (LG-Ex) rats 1 week after surgery. The results are presented as the mean ± SD; *n* = 7 (Normal) and *n* = 28 (LG-Ex) biological replicates. ***p < 0.001. (**c**) Fluorescein staining images and fluorescein infiltration scores of the cornea in Normal and LG-Ex rats one week after surgery. The results are presented as the mean ± SD; *n* = 7 (Normal) and *n* = 28 (LG-Ex) biological replicates. ***p < 0.001. (**d**) Fluorescein staining images and fluorescein infiltration scores of the cornea in Normal, LG-Ex, LG-Ex with ocular instillation of medium (LG-Ex + medium), and LG-Ex with ocular instillation of hAdMSC-CM (LG-Ex + hAdMSC-CM) rats one week after ocular instillation. The results are presented as the mean ± SD; *n* = 7 biological replicates. *p < 0.05. (**e**) Haematoxylin and eosin (H&E) staining images of the cornea of Normal, LG-Ex, LG-Ex + medium, and LG-Ex + hAdMSC-CM rats. Scale bar, 100 µm. *n* = 4 biological replicates. (**f**) Immunostaining images of TJP1 and CDH1 in the cornea of Normal, LG-Ex, LG-Ex + medium, and LG-Ex + hAdMSC-CM rats. Scale bar, 50 µm. *n* = 4 biological replicates. (**g**) Immunostaining images of MUC4 in the cornea of Normal, LG-Ex, LG-Ex + medium, and LG-Ex + hAdMSC-CM rats. Scale bar, 50 µm. *n* = 3 biological replicates.

Finally, we investigated the effects of ocular instillation of hAdMSC-CM on the cornea of LG-Ex rats. 1 week after exorbital and intraorbital lacrimal gland excision of rats, measurements of tear production and fluorescein staining of the cornea were performed, and we confirmed that LG-Ex rats showed decreased tear production and induced corneal epithelial damage, which were similar to clinical DES (Fig. 4a–c). After the induction of corneal epithelial damage, ocular instillation of hAdMSC-CM was administered for one week in LG-Ex rats, and tear production was not affected by hAdMSC-CM (Supplementary Fig. S7a). In contrast, fluorescein staining showed that corneal epithelial damage in LG-Ex rats was suppressed by hAdMSC-CM (Fig. 4d). Furthermore, we investigated the structure of the cornea using haematoxylin and eosin (H&E) staining; the thickness of the corneal epithelium was reduced in LG-Ex rats, and hAdMSC-CM improved thinning of the corneal epithelium (Fig. 4e). To explore the mechanism underlying hAdMSC-CM effects, we examined the inflammation and barrier function of the cornea. H&E staining showed that infiltration of immune-related cells was not observed in the corneas of LG-Ex rats (Fig. 4e). Expression levels of inflammation-related genes, such as *Il6*, *Il1α*, *Tnf*, and *Mmp9* did not increase in the corneas of LG-Ex rats (Supplementary Fig. S7b). Thus, LG-Ex rats did not exhibit inflammation, and epithelial damage to the cornea of LG-Ex rats was not related to inflammation. Immunostaining showed that the expression levels of barrier function-related proteins, such as TJP1, CDH1, and MUC4, were decreased, and localisation of TJP1 was disrupted in the corneal epithelium of LG-Ex rats (Fig. 4f,g). Ocular instillation of hAdMSC-CM increased the expression levels of TJP1, CDH1, and MUC4 and restored the localisation of TJP1 in LG-Ex rats (Fig. 4fg). These results show that ocular instillation of hAdMSC-CM suppressed corneal epithelial damage in LG-Ex rats by improving corneal barrier function.

## Discussion

hAdMSC-CM is expected to serve as a novel therapeutic agent with multiple therapeutic properties^12,13^. However, the effects of hAdMSC-CM on DES have not been sufficiently investigated; in particular, the barrier function has not been explored. In this study, to determine the potential of hAdMSC-CM for the treatment of DES, we evaluated the effects of hAdMSC-CM on BAC-induced cytotoxicity, inflammation, and barrier dysfunction of hCECs and investigated the effects of ocular instillation of hAdMSC-CM on LG-Ex rats.

Our findings showed that hAdMSC-CM suppressed BAC-induced cytotoxicity and inflammation in hCECs (Fig. 1). Expression levels of inflammation-related factors, such as IL6, IL1a, TNF, and MMP9, increased in the tear and cornea of DES, and an increase in these factors contributes to the pathogenesis of DES^25–27^. Inhibition of inflammation is one of the mechanisms of cyclosporine, which is used as a treatment for DES^28^. Therefore, our data suggest that hAdMSC-CM may exert therapeutic effects on DES by suppressing inflammation.

To maintain the barrier function of the cornea, cell junctions, including tight junctions and adherence junctions, of hCECs, are important^29,30^. However, in severe DES, such as Sjogren’s syndrome, cell junctions are disrupted, which impairs the barrier function of the cornea^4,5^. There is no therapeutic agent for DES that improves cell junctions and barrier function of the cornea, and the effects of hAdMSC-CM on the cell junctions and barrier function of the cornea are not known. In this study, for the first time, we found that hAdMSC-CM strengthened cell junctions of hCECs; increased the expression levels of barrier function-related components, such as TJP1, CDH1, and MUC16; and promoted localisation of TJP1 to cell–cell contacts (Fig. 2). TJP1, a component of tight junctions, regulates actin cytoskeleton remodelling and generates the corneal barrier by localising to cell–cell contacts^29^. CDH1 is important for the formation of adherence junctions that maintain cell–cell contacts^31^. Thus, decreased expression level and mislocalisation of TJP1 and CDH1 causes barrier dysfunction of the cornea in DES^4,5,23^. Moreover, MUC16 is a member of the mucin family that plays an important role in forming the mucous barrier of the cornea, and decreased expression level of MUC16 can lead to impaired barrier function of the cornea in DES^32^. Therefore, our findings for the first time showed that hAdMSC-CM may be effective in improving corneal barrier function.

RNA-seq analysis results showed that the effects of hAdMSC-CM may be associated with TGFβ and JAK-STAT signalling pathways (Fig. 3a, Supplementary Fig. S3 and Table 1). Moreover, these signalling pathways are related to inflammation and barrier function in various tissues^33–36^. We found that inhibition of these signalling pathways suppressed inflammation and barrier dysfunction of hCECs, especially inhibition of the TGF-β pathway, contributing significantly to inflammation, showing effects similar to those of hAdMSC-CM (Supplementary Fig. S4 and Fig. 3). Inhibition of the JAK-STAT signalling pathway contributed significantly to barrier function, showing effects similar to those of hAdMSC-CM (Fig. 3). Thus, our findings indicate that hAdMSC-CM exerts multiple effects by inhibiting these signalling pathways. TGFβ increases expression of inflammation related genes via activation of NF-κB signalling pathway^37–40^. Also, we previously reported that inhibition of the TGFβ signalling pathway by hAdMSC-CM suppressed epithelial-mesenchymal transition (EMT) in the cornea^14^. TGFβ leads to EMT through phosphorylation of Smad2/3, resulting in disrupted cell junctions, and which impairs the barrier function of the epithelial cells^41,42^. Subsequently, activation of JAK2/STAT1 and JAK2/STAT3 signalling pathway is associated with inflammation in the corneal epithelial cells of DES model mouse^43,44^. In addition, inhibition of the JAK-STAT signalling pathway is expected to be a useful therapeutic approach in diseases with impaired barrier functions, such as atopic dermatitis and inflammatory bowel disease, by strengthening cell junctions^34–36^. Therefore, our results suggest that inhibitors of TGFβ and JAK-STAT signalling pathways, as well as hAdMSC-CM, may serve as potential therapeutic agents for DES. However, the effects of hAdMSC-CM were not the same as those observed for TGFβ and JAK-STAT signalling pathway inhibition. hAdMSC-derived extracellular vesicles reportedly suppress BAC-induced inflammation by inhibiting the NLRP3 inflammasome^16,17^. Taken together, hAdMSC-CM improved cytotoxicity, inflammation, and barrier dysfunction by regulating multiple signalling pathways, such as TGFβ and JAK-STAT, and additional analysis may reveal other mechanisms underlying the effects of hAdMSC-CM.

In patients with DES, corneal epithelial damage develops as a result of decreased tear production, and the thickness of the corneal epithelium is reduced by friction associated with reduced tear production^45,46^. Our results indicated that LG-Ex rats showed similar characteristics as shown by patients with DES and may be useful for studying DES (Fig. 4b,c). We demonstrated that ocular instillation of hAdMSC-CM suppressed corneal epithelial damage and thinning of the corneal epithelium in LG-Ex rats, suggesting that ocular instillation of hAdMSC-CM may be a potential therapeutic agent for DES (Fig. 4d,e). Moreover, the expression of barrier function-related components, such as TJP1 and CDH1, in the corneal epithelium was disrupted in LG-Ex rats, but it was restored by hAdMSC-CM. The data when correlated with in vitro experimental data, indicated that, ocular instillation of hAdMSC-CM ameliorates barrier dysfunction of the cornea (Figs. 2, 4f). MUC4 is a member of the mucin family and plays an important role in forming the mucous barrier of the cornea. The expression level of MUC4 in the cornea decreases in exorbital lacrimal gland excised mouse models^32,46^. We found that MUC4 expression level decreased in the corneal epithelium of LG-Ex rats, but hAdMSC-CM induced them to increase (Fig. 4g). These results for the first time indicate that ocular instillation of hAdMSC-CM may function as a potential therapeutic agent for DES by improving corneal barrier function. In contrast, we investigated corneal inflammation, but it was not induced in LG-Ex rats (Fig. 4e and Supplementary Fig. S7b). Other groups have reported that ocular instillation of secreted factors from hAdMSCs improved DES in mice by inhibiting inflammation^16,17^. In vitro experiments confirmed that hAdMSC-CM suppressed inflammation in hCECs (Fig. 1d). Therefore, this treatment option may be effective in severe DES with barrier dysfunction and inflammation due to various therapeutic properties. hAdMSC-CM did not affect tear production, but future studies may clarify our understanding and provide additional therapeutic effects caused by indirect action on tissues other than the cornea, such as the conjunctiva. (Supplementary Fig. S7a).

In conclusion, we showed that hAdMSC-CM has many therapeutic properties, which are associated with the TGF-β and JAK-STAT signalling pathways. Ocular instillation of hAdMSC-CM for the treatment of DES suppressed corneal epithelial damage by improving barrier function. Although, LG-Ex animal model shows similar pathology as observed in DES, only in the absence of inflammation, there may exist several inherent differences between the patients with DES and animal models. As one of the approaches to treat severe DES, we established various eye-related tissues, such as the lacrimal gland, corneal epithelial sheet, and conjunctival epithelium, from human pluripotent stem cells, and it may be possible to perform patient transplants using these tissues in the future^47–49^. However, there are difficulties regarding transplantation, including high costs of procedures, the need for advanced technology and facilities, and potential risks to the patients. hAdMSC-CM may be used to address these treatment limitations and is expected to become an innovative therapeutic agent for patients with DES.

## Methods

All of the experiments were performed in accordance with the relevant institutional and national guidelines and regulations.

### Cell culture

hAdMSCs were acquired from PromoCell (Heidelberg, Germany) and cultured in Cellartis MSC Xeno-Free Culture Medium (hMSC culture medium; Takara Bio, Shiga, Japan). To prepare hAdMSC-CM, hAdMSCs were expanded to 70–80% confluence in a T-75 flask. The medium was changed to hMSC or hCEC culture medium (DMEM/F12 (Thermo Fisher Scientific, Tokyo, Japan) containing 2% B-27 supplement (Thermo Fisher Scientific), 20 ng mL^−1^ KGF (Wako, Osaka, Japan), 10 μM Y-27632 (Wako), 100 U mL^−1^ penicillin potassium, and 100 μg mL^−1^ streptomycin sulphate). The supernatant was collected after 72 h of culture, centrifuged at 1500 rpm, and frozen at − 80 °C. hCECs were isolated using an established method and cultured in hCEC culture medium^50^. hCECs were handled according to the tenets of the Declaration of Helsinki. To induce cytotoxicity, inflammation, and barrier dysfunction, BAC (Sigma-Aldrich, Burlington, MA, USA) was administered to hCECs for 10 min. The concentration of BAC was based on previous reports, and most hCECs died after 10 min of treatment at a concentration of 0.01% BAC^22,24^. Therefore, hCECs were treated with 0.0025% BAC or 0.005% BAC for 10 min. Phase-contrast images of hCECs and hAdMSCs were acquired using an EVOS FL Auto system (Thermo Fisher Scientific) and an Axio Observer D1 microscope (Carl Zeiss, Jena, Germany).

### Flow cytometry

The hAdMSCs were collected using Accutase (Thermo Fisher Scientific). Dissociated cells were stained with PE-conjugated anti-CD29 (555,443, BD Biosciences, San Jose, CA, USA), PE-conjugated anti-CD34 (343,506, BioLegend, San Diego, CA, USA), PE-conjugated anti-CD45 (304,008, BioLegend), PE-conjugated anti-CD73 (550,257, BD Biosciences), PE-conjugated anti-CD90 (IM1840U Beckman Coulter, Brea, CA, USA), and PE-conjugated anti-CD105 (560,839, BD Biosciences) for 1 h on ice (Table 2). Flow cytometry was performed using a SH800 cell sorter (Sony Biotechnology, Tokyo, Japan). The flow cytometry results were analysed using SH800 and FlowJo v10.8 Software (BD Biosciences).

**Table 2:** Details of antibodies used for immunostaining, western blotting and flow cytometry.

Antigen	Identifier	Supplier	Dilution
CD29	Mouse monoclonal; P5D2 PE-conjugated	R&D systems	1:500
CD34	Mouse monoclonal; 581 PE-conjugated	BioLegend	1:500
CD45	Mouse monoclonal; HE30 PE-conjugated	BioLegend	1:500
CD73	Mouse monoclonal; AD2 PE-conjugated	BD Biosciences	1:500
CD90	Mouse monoclonal; F15-42-1-5 PE-conjugated	Beckman Coulter	1:500
CD105	Mouse monoclonal; SN6/N1—3A1 PE-conjugated	Adipogen	1:500
ZO-1	Mouse monoclonal; 1A12	Thermo Fisher Scientific	1:200
CDH1	Rabbit polyclonal	Atlas Antibodies	1:200
MUC16	Mouse monoclonal; OC125	Abcam	1:200
MUC4	Mouse monoclonal; 1G8	Thermo Fisher Scientific	1:250
α-Tubulin	Rabbit polyclonal	Cell signaling technology	1:2000
Smad2/3	Rabbit polyclonal	Cell signaling technology	1:1000
Phospho-Smad2 (Ser465/467)	Rabbit polyclonal	Cell signaling technology	1:1000
Jak2	Rabbit monoclonal; D2E12	Cell signaling technology	1:1000
Phospho-Jak2 (Tyr1007/1008)	Rabbit monoclonal; C80C3	Cell signaling technology	1:1000
STAT1	Rabbit monoclonal; D1K9Y	Cell signaling technology	1:1000
Phospho-Stat1 (Tyr701)	Rabbit monoclonal; D4A7	Cell signaling technology	1:1000

### Cell viability assay

hCECs were seeded at 9 × 10^3^ cells/well in 24-well plates. After a 24 h culture, the medium was changed as follows: 1. hMSC culture medium or hAdMSC-CM derived from hMSC culture medium (hAdMSC-CM (m)), 2. hCEC culture medium or hAdMSC-CM derived from hCEC culture medium. Following another 24 h culture, hCECs were cultured in hCEC culture medium containing 0.0025–0.0050% BAC for 10 min, and the medium was then changed as described above. After 48 h of culture, the medium was replaced with hCEC culture medium containing 10% AlamarBlue Cell Viability Reagent (Thermo Fisher Scientific) and incubated at 37 °C for 2 h. Absorbance (Abs) was read using ARVO X4 (PerkinElmer, Waltham, MA, USA) according to the manufacturer’s instructions (Thermo Fisher).

### Quantitative real-time reverse transcription PCR (qRT-PCR)

The mRNA expression of hCECs and rat corneas was analysed using qRT-PCR. The rat cornea was pulverised using the TissueLyser II (Qiagen, Venlo, Netherlands) system. Total RNA was extracted using QIAzol Lysis Reagent (Qiagen), and cDNA was synthesised using the SuperScript III First-Strand Synthesis System (Thermo Fisher Scientific). Quantitative PCR was performed using the ABI PRISM 7500 Fast Sequence Detection System (Thermo Fisher Scientific), according to the manufacturer’s instructions. The primers used in this study are listed in Table 3.

**Table 3:** List of qRT-PCR primers.

Target	Species	Forward	Reverse
*GAPDH*	Human	GGAGCGAGATCCCTCCAAAAT	GGCTGTTGTCATACTTCTCATGG
*IL6*	Human	TGGCAGAAAACAACCTGAACC	GGCTTGTTCCTCACTACTCTCA
*TNF*	Human	CATCTTCTCGAACCCCGAGT	ATGAGGTACAGGCCCTCTGAT
*IL1A*	Human	CAGCCAGAGAGGGAGTCATTT	TGTCTGGAACTTTGGCCATCTT
*MMP9*	Human	CGACGTCTTCCAGTACCGA	TTCAACTCACTCCGGGAACTC
*TJP1*	Human	GGGACAACAGCATCCTTCCA	ATCACAGTGTGGTAAGCGCA
*CLDN1*	Human	CTGTCATTGGGGGTGCGATA	CTGGCATTGACTGGGGTCAT
*CDH1*	Human	CCTGGGACTCCACCTACAGA	TGGATTCCAGAAACGGAGGC
*MUC4*	Human	GCAAGCATCGGACTTCACAC	GCTTCAATCACACGACCACC
*MUC16*	Human	ACGGTTACAATGAACCTGGTC	GTGTGAGGGTCTTCAGGTGG
*Gapdh*	Rat	TGCACCACCAACTGCTTAGC	GGCATGGACTGTGGTCATGAG
*Il6*	Rat	GCGATGATGCACTGTCAGAA	CGGAACTCCAGAAGACCAGAG
*Tnf*	Rat	ATGGGCTCCCTCTCATCAGT	GGGCTTGTCACTCGAGTTTTG
*Il1a*	Rat	TCGGGAGGAGACGACTCTAA	GGTCGGTCTCACTACCTGTG
*Mmp9*	Rat	GATCCCCAGAGCGTTACTCG	GTTGTGGAAACTCACACGCC

### Transepithelial Electrical Resistance (TER)

hCECs were seeded at 2.5 × 10^4^ cells/insert in 12-well cell culture inserts. After a 24 h culture, the medium was changed to hCEC culture medium or hAdMSC-CM derived from hCEC culture medium for the apical chamber. For the basal chamber, the medium was changed to hCEC culture medium without KGF and Y-27632. Following another 24 h culture, the medium was replaced with hCEC culture medium (containing 0.0025% BAC for the apical chamber) for 10 min. Then, for both the apical and basal chambers, the medium was changed as mentioned above. After 24 h of culture, TER was measured using MilliCell ERS-2 (Millipore, Billerica, MA, USA) according to the manufacturer’s instructions.

### Immunofluorescence staining

The hCECs and rat corneas were fixed in 4% paraformaldehyde (Wako). Samples were washed three times with Tris-buffered saline (TBS; Takara Bio) and incubated with TBS containing 5% normal donkey serum (Jackson ImmunoResearch, Bar Harbor, Maine, USA) and 0.3% Triton X-100 (Sigma-Aldrich, St. Louis, MO, USA) for 1 h to block non-specific reactions. Subsequently, they were incubated with the primary antibodies listed in Table 2 at 4 °C for 24 h. Samples were then washed three times with TBS and stained with Alexa Fluor 488- and AF568-conjugated secondary antibodies (Thermo Fisher Scientific) and Hoechst 33,342 (Thermo Fisher Scientific) at room temperature for 1 h. Thereafter, the stained samples were washed with TBS three times and observed under a fluorescence microscope FV3000 (Olympus, Tokyo, Japan).

### RNA sequencing analysis

cDNA library preparation from RNA and analyses were performed using a pipeline provided by Rhelixa, Inc. Briefly, the NEBNext Poly(A) mRNA Magnetic Isolation Module and NEBNext Ultra II Directional RNA Library Prep Kit were used for cDNA library preparation, and the Illumina NovaSeq 6000 system was used for sequencing in the 150-bases paired-end mode. FastQC v.0.11.7, was used for a quality check of the sequencing run, and Trimmomatic v.0.38 was used to trim the sequenced reads. HISAT2 v.2.1.0. was used to map the sequenced reads to the human reference genome sequences (hg38). The number of raw reads mapped to the exon regions was calculated using FeatureCounts v.1.6.3. The analysis of differentially expressed genes (DEGs) and heat mapping were performed using integrated Differential Expression and Pathway analysis (iDEP v.0.95).

### Western blot analysis

Whole cell lysates were extracted using RIPA buffer containing a proteinase inhibitor cocktail (Nacalai Tesque, Kyoto, Japan) and PhosSTOP (Roche, Basel, Switzerland). Protein concentrations were measured using the Pierce BCA Protein Assay Kit (Thermo Fisher Scientific). Protein samples (25 µg) were loaded on NuPAGE 4–12% Bis–Tris gels (Thermo Fisher Scientific) and transferred to polyvinylidene fluoride membranes (GE Healthcare, Chicago, IL, US). Thereafter, membranes were blocked with 5% bovine serum albumin (Sigma) or 5% skim milk (Wako) in TBS with Tween-20 (TBST; Takara Bio) for 1 h. Next, membranes were incubated with the primary antibodies listed in Table 2 at 4 °C for 24 h. Subsequently, the membranes were incubated with HRP-conjugated secondary antibodies (1:5000; GE Healthcare) at RT for 1 h, and then detected with ECL Select Western Blotting Detection Reagent (GE Healthcare). Protein bands were visualised using a Molecular Imager ChemiDoc XRS + system (Bio-Rad Laboratories, Hercules, CA, USA). For stripping the protein bands, membranes were incubated at 50 °C for 30 min in stripping buffer, which was composed of 10% sodium dodecyl sulphate (Wako), 0.5 M tris (hydroxymethyl) aminomethane hydrochloride (Nacalai Tesque), and 100 mM 2-Mercaptoethanol (Sigma). Membranes were then blocked and incubated with subsequent antibodies.

### Signalling pathway inhibitor experiments

SB431542 (SB; Cayman Chemical, Ann Arbor, MI, US) and ruxolitinib (ru; Cayman Chemical) were dissolved in dimethyl sulfoxide (Wako) as stock solutions. hCECs were treated with SB431542 (1, 10, and 30 µM) or ruxolitinib (0.3, 3, and 30 µM). Next, hCECs were analysed for viability, inflammation, and barrier function.

### TUNEL assay

Apoptotic cells were detected using the TUNEL Apoptosis Detection Kit (Roche). Briefly, hCECs were fixed in 2% paraformaldehyde (Wako). Samples were washed three times with TBS and incubated with 0.1% Trisodium Citrate (Wako) and 0.3% Triton X-100 (Sigma-Aldrich, St. Louis, MO, USA) for 2 min to block non-specific reactions. Intracellular DNA fragments were labeled using the TUNEL reaction mixture and stained with Hoechst 33342 at room temperature for 1 h. The stained samples were then washed with TBS thrice and observed under a FV3000 fluorescence microscope.

### Exorbital and intraorbital lacrimal gland excision

All animal experiments were performed in accordance with ARRIVE guidelines and the ARVO Statement for the Use of Animals in Ophthalmic and Vision Research and were approved by the animal ethics committee of Osaka University (certificate number 02–024-000). Male Slc:SD rats (body weight 200–250 g, aged 7 weeks, SLC, Inc., Tokyo, Japan) were anaesthetised with an intraperitoneal injection of 0.15 mg kg^−1^ medetomidine hydrochloride (Nippon Zenyaku Kogyo, Fukui, Japan), 2.0 mg kg^−1^ midazolam (Maruishi Pharmaceutical, Osaka, Japan) and 2.5 mg kg^−1^ butorphanol tartrate (Meiji Seika Pharma, Tokyo, Japan). Under deep anaesthesia, the exorbital and intraorbital lacrimal glands were excised only on the left side, after which the incisions were sutured with an 8–0 nylon thread using a stereomicroscope (Olympus). After surgery, 0.3% ofloxacin ointment (Santen Pharmaceutical, Osaka, Japan) and 0.1% betamethasone sodium phosphate ointment (Shionogi Pharmaceutical) were administered twice daily.

### Ocular instillation of hAdMSC-CM, measurements of tear production, and fluorescein stain scoring

One week after exorbital and intraorbital lacrimal gland excision, the rats were anaesthetised with 3% inhalant isoflurane, and tear production was measured using Zone-Quick (Showa Yakuhin, Tokyo, Japan) for 10 s. Next, the rat cornea was stained with 2 µL 0.5% fluorescein for 1 min and washed five times with 1 mL Sterile Saline (Otsuka Pharmaceutical Factory, Tokushima, Japan). The corneal epithelial damage grade was evaluated using a slit-lamp microscope (Carl Zeiss), and fluorescein stain scoring was assessed. The cornea was divided into four quadrants, and each quadrant score was evaluated individually. Each quadrant was scored from 1 to 5: 1 = no staining; 2 = weak, fluorescence is partially dotted; 3 = weak, fluorescence is scattered throughout; 4 = strong, fluorescence is scattered throughout; and 5 = strong, fluorescence is scattered without gaps. The scores of the four quadrants were summed to arrive at the final score (minimum = 4, maximum = 20). After the rats with the highest and lowest fluorescein staining scores were excluded, they were randomly divided into four groups (1. Normal; 2. LG-Ex; 3. LG-Ex + ocular instillation for hCEC culture medium; 4. LG-Ex + ocular instillation of hAdMSC-CM). After grouping, 10 µL of hCEC culture medium or hAdMSC-CM was applied to the left eye three times per day for one week, and tear production measurement and fluorescein staining were conducted under anaesthesia. Finally, all rats were sacrificed and the cornea was used for qRT-PCR, immunofluorescence staining, and H&E staining.

### H&E staining

The rat corneas were fixed with 10% formaldehyde neutral buffer solution (Nacalai Tesque) and paraffin (Thermo Fisher Scientific) using an ASP6025 Tissue Processor (Leica, Wetzlar, Germany). Samples were cut into 8 µm sections using a SM2010R microtome (Leica) and stained with H&E (Sakura Finetek Japan, Tokyo, Japan). Subsequently, the sections were imaged using an Axio Observer Z1, D1 (Carl Zeiss).

### Statistical analysis

All data are presented as mean ± SD. The Student’s *t*-test was performed for two-group comparisons of parametric data (Fig. 4c). The Mann–Whitney U test was performed for two-group comparisons using non-parametric data (Fig. 4b). The Tukey–Kramer test was performed for multiple comparisons of parametric data (Fig. 1c, d, 2b,g, 3c,f, and Supplementary Fig. S4b, S4c). Steel–Dwass tests were performed for multiple comparisons in non-parametric data (Fig. 4d and Supplementary Fig. S7a, S7b). All statistical analyses were performed using Bell Curve for Excel v.3.20 (Social Survey Research Information Co., Ltd., Tokyo, Japan). All statistical analyses were conducted with a significance level of a = 0.05 (*p* < 0.05).

### Supplementary Information

Supplementary Figures.

## Data Availability

RNA-seq datasets have been deposited at the NCBI GEO repository under accession number GSE225408. Reviewer's token number is “uturocsojtiffwt” to access GEO accession GSE225408. Source data are provided with this paper.
