# Supplementary material for: Ocular instillation of conditioned medium from mesenchymal stem cells is effective for dry eye syndrome by improving corneal barrier function
Source: Sci Rep. 2023 Aug 11;13:13100. doi: 10.1038/s41598-023-40136-2 (PMC10421917; doi:10.1038/s41598-023-40136-2)
Supplement: Supplementary file 1 — Supplementary Figures. [file 41598_2023_40136_MOESM1_ESM.pdf]

Supplementary Figures 1-7

Ocular instillation of conditioned medium from mesenchymal stem cells is effective for dry eye syndrome by improving corneal barrier function

Tsutomu Imaizumi, Ryuhei Hayashi, Yuji Kudo, Xiaoqin Li, Kaito Yamaguchi,
Shun Shibata, Toru Okubo, Tsuyoshi Ishii, Yoichi Honma, and Kohji Nishida

Supplementary Figure S1

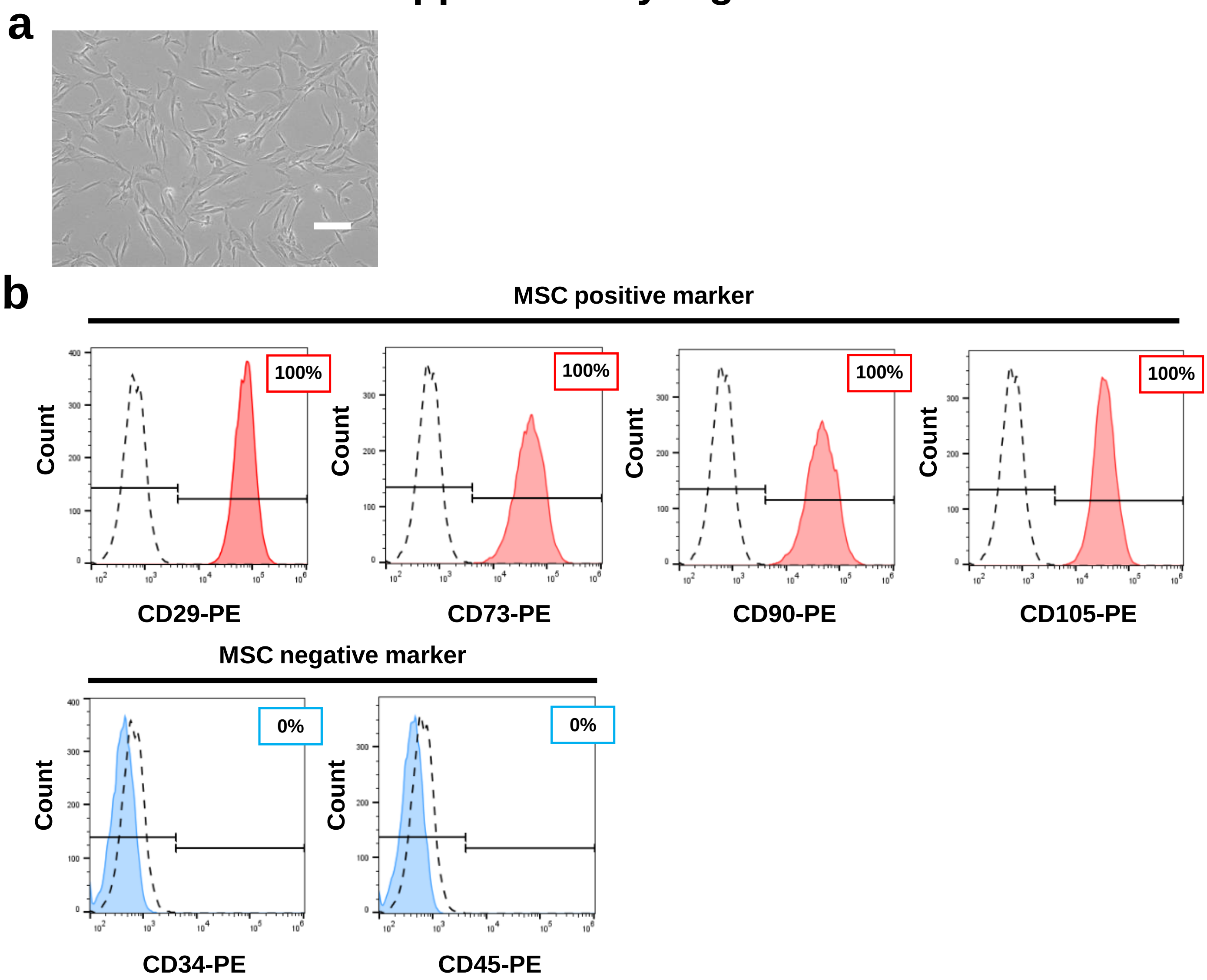

Supplementary Figure S1. Characteristics of hAdMSCs. (a) Phase images of hAdMSCs. Scale bar, 200 µm. *n* = 12 biological replicates. (b) Expression of hMSC positive and negative markers. *n* = 3 biological replicates. hAdMSCs; human adipose-derived mesenchymal stem cells.

Supplementary Figure S2

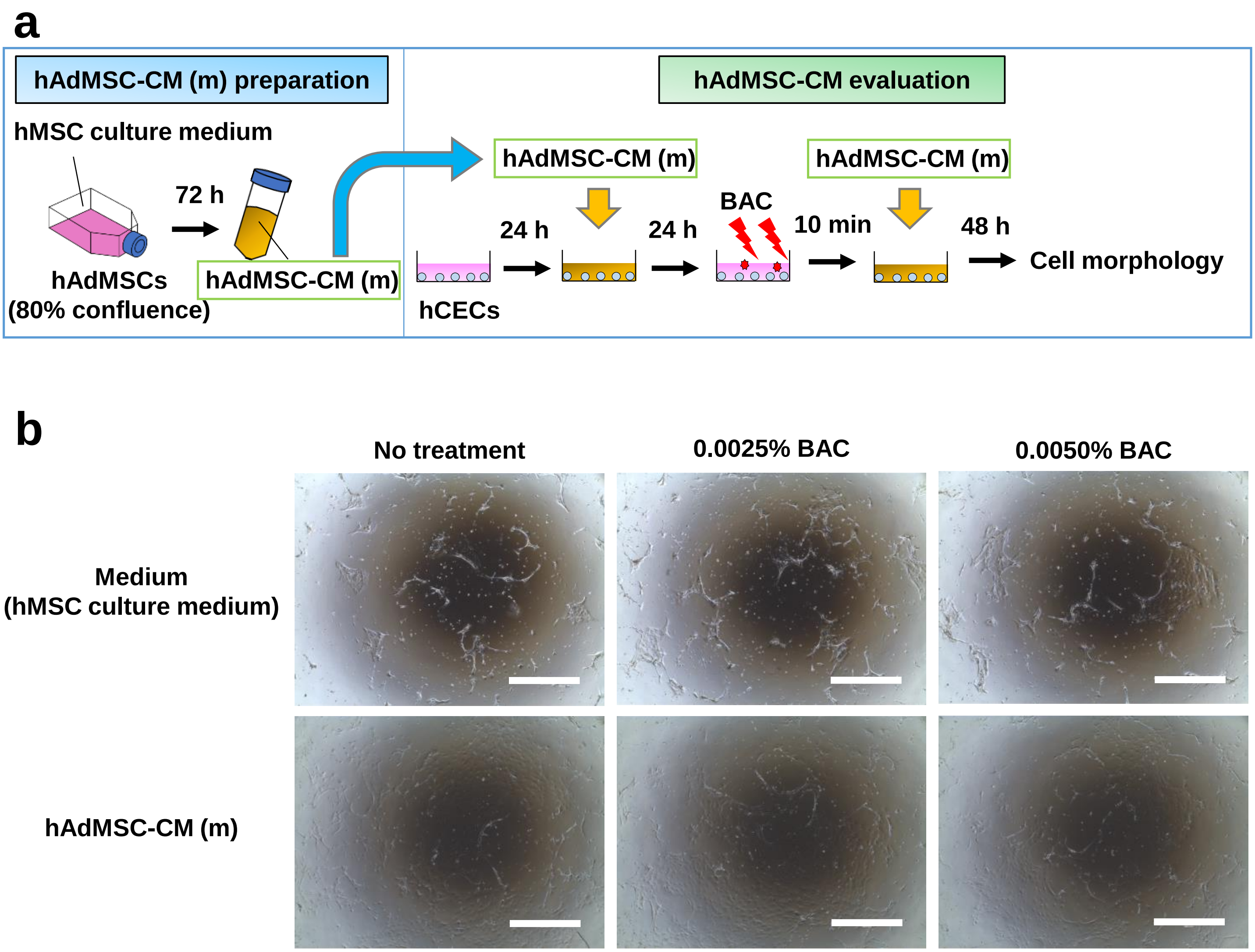

Supplementary Figure S2. Morphology of hCECs could not be maintained by hAdMSC-CM derived from hMSC culture medium. (a) Schematic representation of hAdMSC-CM preparation using hMSC culture medium (hAdMSC-CM (m))and the evaluation of hAdMSC-CM (m) on hCECs. (b) Phase images of hCECs 2 days after treatment with or without BAC. Scale bar, 1000 µm. *n* = 9 biological replicates. hAdMSC-CM; conditioned medium of human adipose-derived mesenchymal stem cells, BAC; benzalkonium chloride, hCECs; human corneal epithelial cells.

Supplementary Figure S3

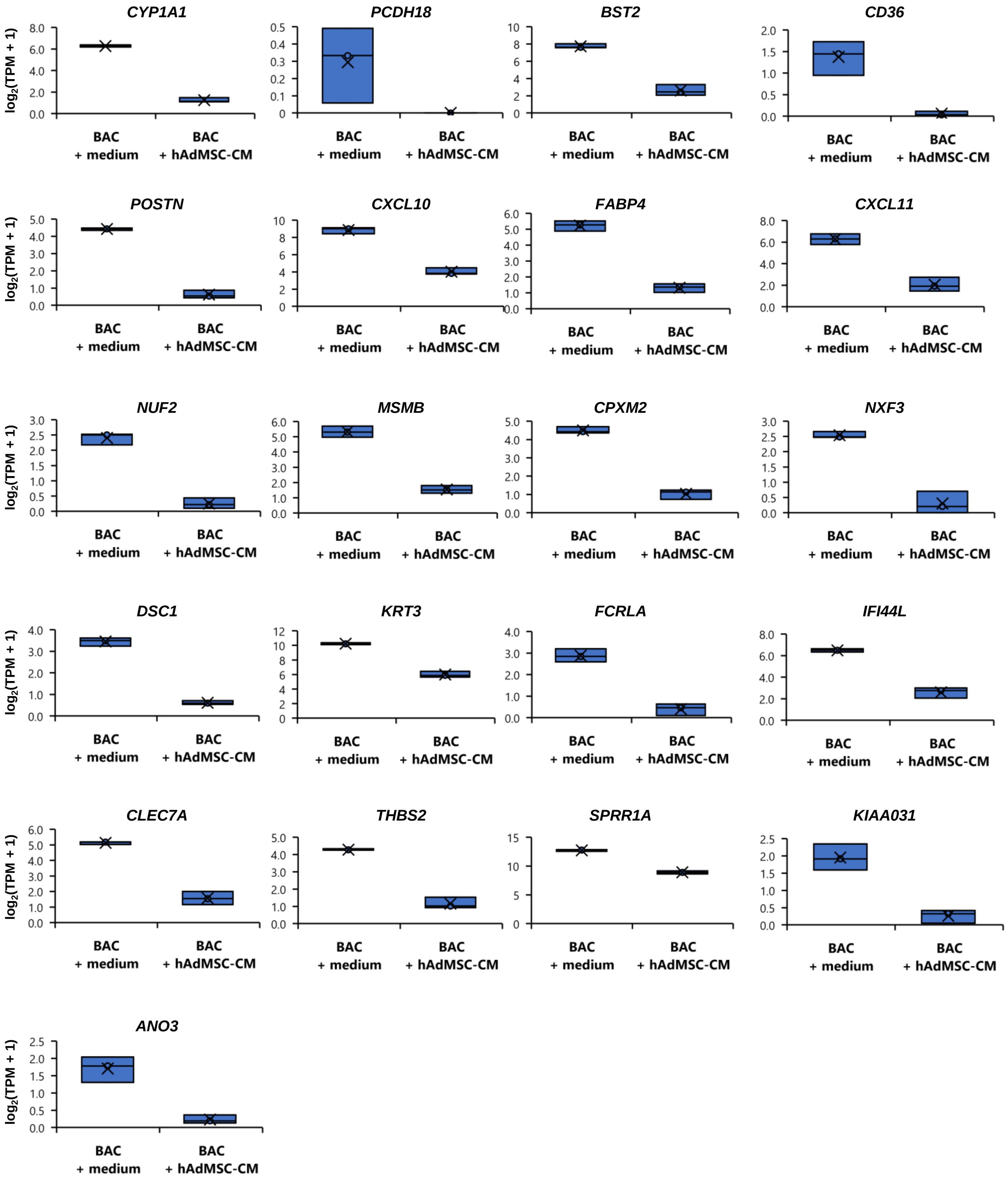

Supplementary Figure S3. Expressions levels of TGFβ or JAK-STAT signalling pathways-related genes. Box plots of TGFβ or JAK-STAT signalling pathways-related genes in the top 30 genes with significant fold reductions in expression levels due to hAdMSC-CM. *n* = 3 biological replicates.

Supplementary Figure S4

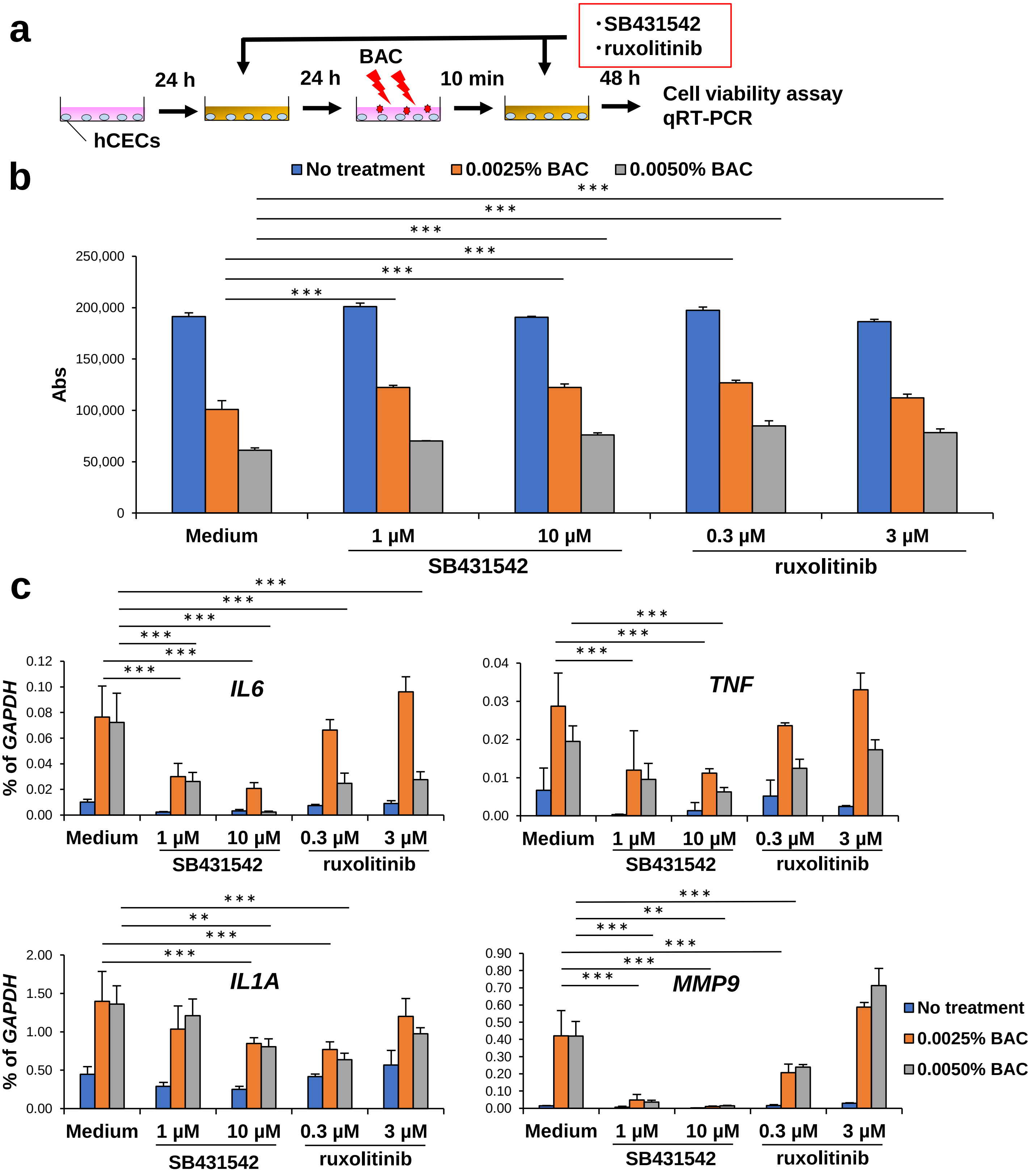

Supplementary Figure S4. BAC-induced cytotoxicity and inflammation of hCECs are suppressed by inhibition of TGFβ and JAK-STAT signalling pathways. (a) Schematic representation of the evaluation of SB431542 and ruxolitinib on BAC-induced cytotoxicity and inflammation of hCECs. (b) Cell viability assay of hCECs 2 days after treatment with or without BAC. The results are presented as the mean ± SD; n = 4 biological replicates. ***$p < 0.001$. (c) Expression levels of inflammation-related genes in hCECs 2 days after treatment with or without BAC. The results are presented as the mean ± SD; n = 4 biological replicates. **$p < 0.01$, and ***$p < 0.001$.

Supplementary Figure S5

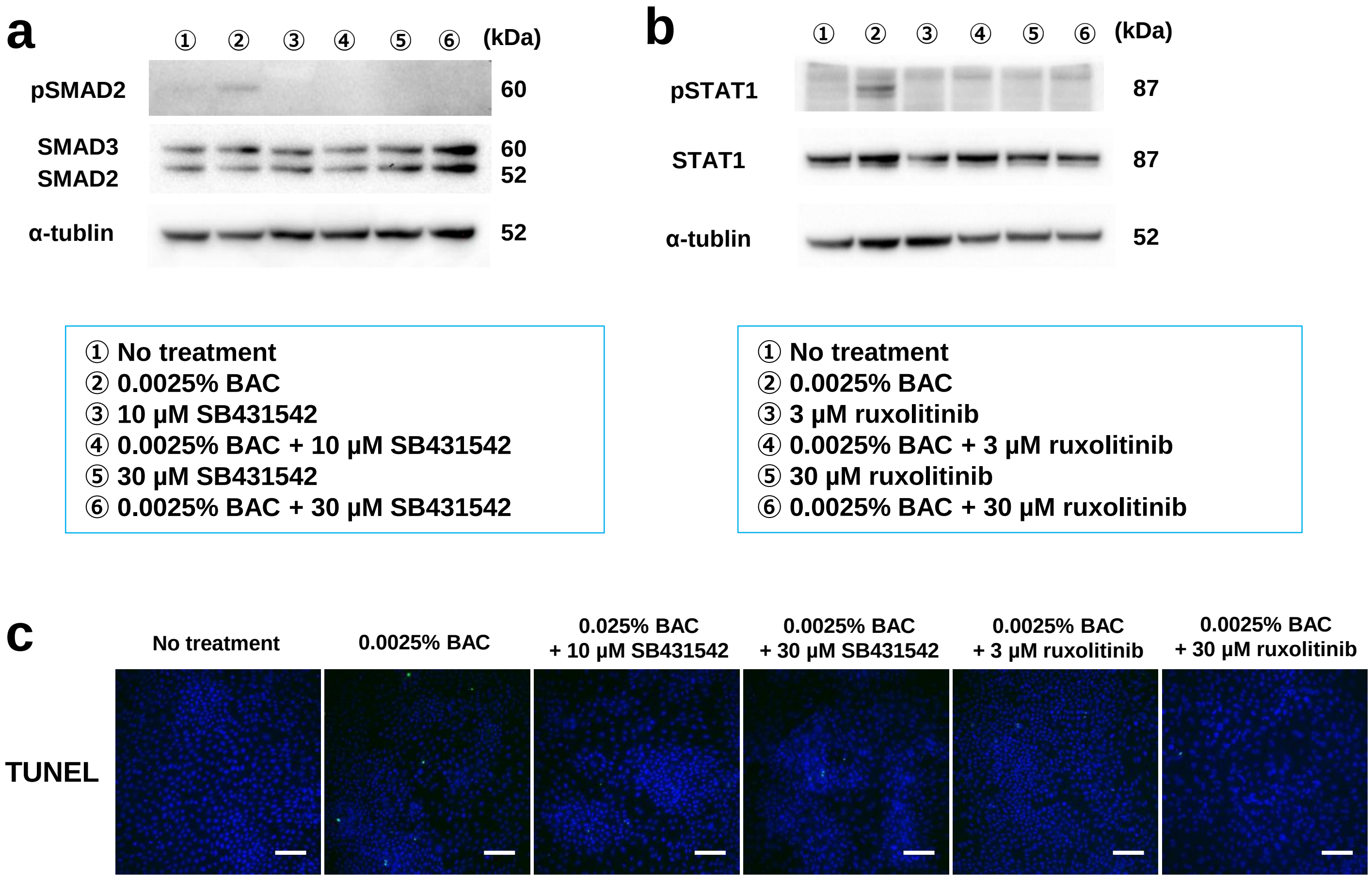

Supplementary Figure S5. Number of apoptotic cells was not increased by inhibition of TGFβ and JAK-STAT signalling pathways. (a) Expression of TGFβ signalling-related proteins. *n* = 3 biological replicates. (b) Expression of JAK-STAT signalling-related proteins. *n* = 3 biological replicates. (c) Fluorescein staining images of TUNEL in hCECs 1 day after treatment with or without BAC. Scale bar, 100 μm. *n* = 4 biological replicates. TUNEL; Terminal deoxynucleotidyl transferase dUTP nick end labelling.

Supplementary Figure S6

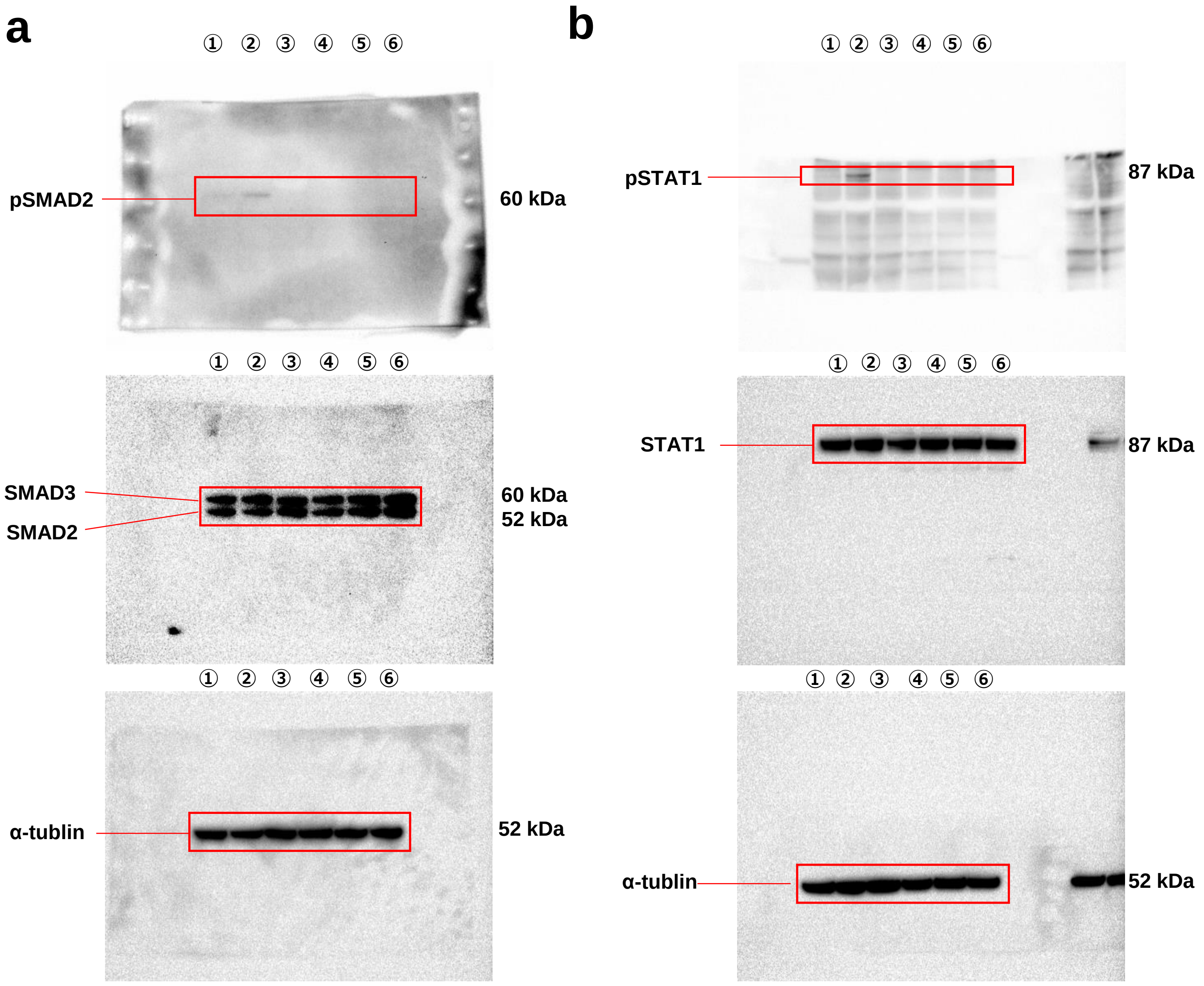

a

① No treatment
② 0.0025% BAC
③ 10 μM SB431542
④ 0.0025% BAC + 10 μM SB431542
⑤ 30 μM SB431542
⑥ 0.0025% BAC + 30 μM SB431542

b

① No treatment
② 0.0025% BAC
③ 3 μM ruxolitinib
④ 0.0025% BAC + 3 μM ruxolitinib
⑤ 30 μM ruxolitinib
⑥ 0.0025% BAC + 30 μM ruxolitinib

Supplementary Figure S6. Uncropped western blots used for the preparation of supplementary Figure S5. (a) Expression of TGFβ signalling-related proteins. *n* = 3 biological replicates. (b) Expression of JAK-STAT signalling-related proteins. *n* = 3 biological replicates.

Supplementary Figure S7

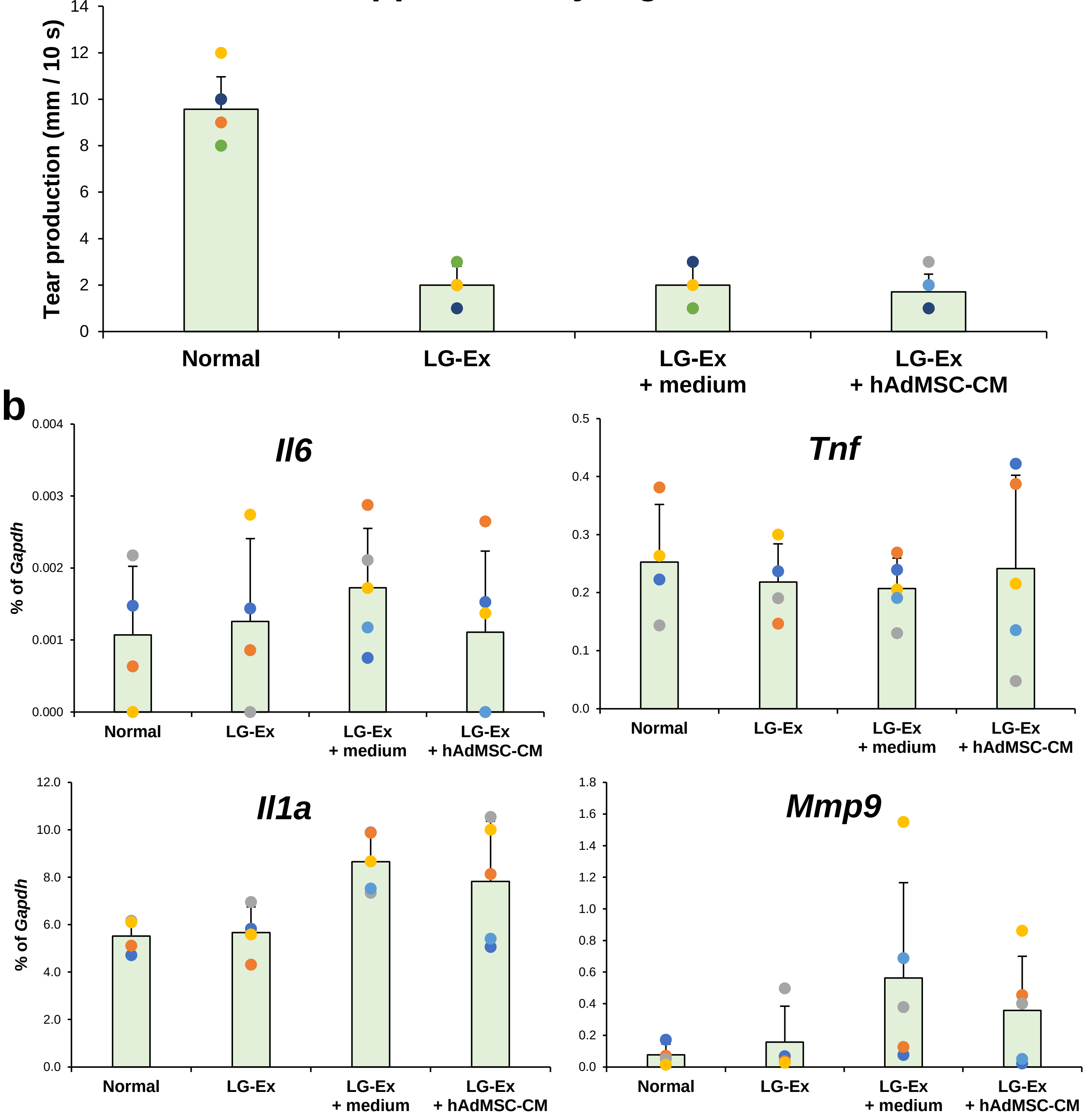

Supplementary Figure S7. Examination of tear production and corneal inflammation in LG-Ex rats. (a) Measurement of tear production in SD (Normal), LG-Ex, LG-Ex with ocular instillation of medium (LG-Ex + medium), and LG-Ex with ocular instillation of hAdMSC-CM (LG-Ex + hAdMSC-CM) rats 1 week after ocular instillation. The results are presented as the mean ± SD; *n* = 7 biological replicates. (b) Expression levels of inflammation related-genes in the cornea of Normal, LG-Ex, LG-Ex + medium, and LG-Ex + hAdMSC-CM rats. The results are presented as the mean ± SD; *n* = 4 (Normal and LG-Ex) and n = 5 (LG-Ex + medium and LG-Ex + hAdMSC-CM) biological replicates.
